# Frailty and Recurrent Cardiovascular Events in Patients With Obstructive Sleep Apnoea: The SAVE Study

**DOI:** 10.1002/jcsm.70252

**Published:** 2026-03-19

**Authors:** Shoujiang You, Danni Zheng, Katie Harris, Kelly A. Loffler, R. Doug McEvoy, Ruth Peters, Qiang Li, Ferran Barbé, Linan Chen, Xiaoying Chen, Yongjun Cao, Chun‐Feng Liu, Geraldo Lorenzi‐Filho, Mark Woodward, John Chalmers, Craig S. Anderson, the SAVE Investigators

**Affiliations:** ^1^ Department of Neurology and Clinical Research Center of Neurological Disease The Second Affiliated Hospital of Soochow University Suzhou China; ^2^ The George Institute for Global Health, Faculty of Medicine University of New South Wales Sydney Australia; ^3^ Flinders Health and Medical Research Institute, College of Medicine and Public Health Flinders University Adelaide Australia; ^4^ Respiratory Department Centro de Investigación Biomédica en Red de Enfermedades Respiratorias, Institut de Recerca Biomédica de Lleida Madrid Spain; ^5^ Instituto do Coracao (Incor) and Hospital Universitario Sao Paulo Brazil; ^6^ The George Institute for Global Health, School of Public Health Imperial College London London UK; ^7^ The Institute of Science and Technology for Brain‐Inspired Research Fudan University Shanghai China; ^8^ Neurology Department Royal Prince Alfred Hospital Sydney Australia

**Keywords:** frailty, obstructive sleep apnoea, recurrent cardiovascular events, risk prediction, SAVE

## Abstract

**Background:**

Frailty is a common syndrome in patients with cardiovascular disease (CVD). Whether frailty modifies the risk of recurrent cardiovascular events in patients with established CVD and obstructive sleep apnoea (OSA) is uncertain. We aimed to determine associations of frailty and the risk of recurrent cardiovascular events in adults with moderate–severe OSA and established CVD.

**Methods:**

Post hoc analyses of the international Sleep Apnea Cardiovascular Endpoints (SAVE) trial where participants from 89 clinical centres in seven countries with moderate‐to‐severe OSA and established CVD were randomised to usual care plus continuous positive airway pressure (CPAP) treatment or usual care alone. Participants were categorised using the Rockwood frailty index (FI) into three groups: nonfrail (FI ≤ 0.210), moderately frail (FI 0.211–0.310) and severely frail (FI ≥ 0.311). Cox proportional hazards models were used to assess associations of FI and both composite and individual cardiovascular outcomes over an average follow‐up period of 3.7 years.

**Results:**

There were 2653 OSA participants (mean age 61.3 [SD 7.8] years, and 507 [19.1%] were female) with established CVD included with a mean FI of 0.290 (SD 0.125). There were 783 (29.5%) and 1006 (37.9%) classified as moderately and severely frail, respectively. Compared to those without frailty, those with severe frailty had increased risks of the composite cardiovascular endpoint (hazard ratio [HR], 2.41; 95% confidence interval [CI], 1.88–3.11), and separately for stroke (HR, 2.40; 95% CI, 1.54–3.74), hospitalisation for unstable angina (HR, 2.94; 95% CI, 1.98–4.35), all‐cause mortality (HR, 1.77; 95% CI, 1.01–3.11) and CVD death (HR, 2.51; 95% CI, 1.13–5.60), during a mean 3.7 years of follow‐up. There was a similar level of adherence to CPAP treatment (*p* = 0.488) across baseline frailty groups and no heterogeneity in the effect of CPAP treatment on composite and separate cardiovascular events.

**Conclusions:**

In the SAVE cohort of adults with co‐occurring OSA and CVD, a higher FI was associated with significantly higher risk of recurrent cardiovascular events. Frailty did not modify CPAP treatment adherence or the effect of CPAP on recurrent cardiovascular events.

Trial Registration: ClinicalTrials.gov identifier: NCT00738179.

**Abbreviations**
AHI: apnoea–hypopnea index
CVD: cardiovascular disease
CPAP: continuous positive airway pressure
ESS: Epworth Sleepiness Scale
FI: frailty index
OSA: obstructive sleep apnoea
ODI: oxygen desaturation index
SAVE: Sleep Apnea Cardiovascular Endpoints
SF‐36: 36‐Item Short Form Health Survey
TIA: transient ischemic attack

## 1 Introduction

Frailty is increasingly recognised as a common syndrome associated with aging, characterised by reduced physiological reserve, dysregulation across multiple organ systems and impaired homeostatic tolerance to stressors [1, 2]. In cardiovascular medicine, frailty has emerged as a powerful predictor of adverse outcomes and a complicating factor in disease management, treatment response and prognosis in older adults with cardiovascular disease (CVD) [3, 4, 5]. Obstructive sleep apnoea (OSA) is a global health problem, characterised by upper airway occlusion during sleep, leading to intermittent hypoxia, sleep fragmentation and intrathoracic pressure changes [6]. OSA is common in people with established CVD [7, 8, 9], and significantly increases the risk of incidents and recurrent cardiovascular events [7, 8, 9, 10, 11, 12].

Having a high frailty index (FI) is associated with an increased risk of falls, immobility, impaired quality of life and mortality [13, 14]. Frailty, OSA and CVD often coexist [7, 8, 15, 16, 17], with a high FI associated with an incremental increase in the risk of CVD in the general population [14, 18, 19]. However, little is known about the strength of the association between frailty and recurrent cardiovascular events in the increasingly prevalent high‐risk individuals with co‐occurring OSA and established CVD.

Some studies suggest that frailty may lead to poor treatment adherence, reduced tolerance and diminished effectiveness of interventions [20, 21]. Conversely, other studies indicate that frail patients experience equivalent or even enhanced benefits of intervention treatments as compared to nonfrail patients [22, 23]. The international Sleep Apnea Cardiovascular Endpoints (SAVE) trial showed that continuous positive airway pressure (CPAP) plus usual care did not prevent major cardiovascular events in adults with moderate–severe OSA and established CVD, compared with usual care alone [24]. Lifestyle interventions and CPAP therapy are the first‐line treatments for OSA [25]. However, whether frailty influences CPAP adherence and modifies the effects of CPAP treatment on recurrent cardiovascular events in patients with OSA and established CVD is unknown.

We hypothesised that having higher baseline FI would be associated with an increased risk of recurrent cardiovascular events and may adversely affect CPAP adherence, thereby modifying the treatment effect of CPAP in patients with OSA and established CVD. To test this hypothesis, we conducted a post hoc analysis of the SAVE database to determine associations of FI and the risk of its primary composite cardiovascular endpoint and individual major cardiovascular events. We also examined whether frailty modifies the effect of CPAP treatment on these outcomes and influences CPAP adherence.

## 2 Methods

### 2.1 Design

SAVE was an international, multicentre, randomised, parallel‐group, open‐label, blinded endpoint‐assessed clinical trial that determined the effectiveness of CPAP on top of standard of care versus standard care alone to reduce recurrent cardiovascular events in high‐risk adults with OSA [24, 26]. In brief, a total of 2687 adults (aged 45 to 75 years) with a diagnosis of moderate‐to‐severe OSA and established coronary artery disease and/or cerebrovascular disease were randomised to receive CPAP plus usual care, or usual care alone, at 89 clinical centres in seven countries from December 2008 to November 2013, with follow‐up until the end of 2015 (average follow‐up period of 3.7 years). Randomisation was performed using the minimisation procedure to balance the group assignments according to site and type of CVD. The details of inclusion and exclusion criteria were based on the original trial design [24, 26]. In brief, the diagnosis of OSA was based on a moderate‐to‐high oxygen desaturation index (ODI, ≥12 events per hour with ≥4% oxygen saturation dip), established using a home sleep screening device (ApneaLink, ResMed) confirmed centrally by sleep specialists. People were excluded if they had severe daytime sleepiness (Epworth Sleepiness Scale [ESS] score >15), very severe nocturnal hypoxemia (oxygen saturation <80% for >10% of the monitoring time) or a predominantly Cheyne–Stokes respiration pattern. The study is registered at ClinicalTrials.gov (NCT00738179). This article followed the STROBE (Strengthening the Reporting of Observational Studies in Epidemiology) reporting guidelines [27].

### 2.2 FI Calculation

We used the Rockwood accumulation of deficits approach to construct a 31‐item FI based on data being available for at least 30 items of health deficit covering symptoms, signs, diseases and functional limitations pertaining to physiological systems and multiple domains that are associated with health and not simply a part of normal aging, which has been extensively validated in large clinical trial and population‐based cohorts [13, 18, 28]. Our FI was derived from participant data on comorbidities (medical history), vital signs, lifestyle factors, OSA severity measures and eight domains of the 36‐Item Short Form Health Survey (SF‐36) at baseline based on clinical relevance, available data in the SAVE study and prior literature [5, 13, 14, 18, 22, 28], as detailed in Table S1. Each variable was recoded on a scale from 0 to 1, with 0 representing no deficit and 1 representing total deficit. Binary variables, such as baseline comorbidities, were scored 0 (absent) or 1 (present). Ordinal variables were scaled from 0 to 1, with 1 representing the greatest severity. Continuous variables, including vital signs, OSA severity measures and eight domains of SF‐36, were categorised based on clinical thresholds or centiles and scored as 0 (normal) or 1 (abnormal). After excluding 34 participants with data for fewer than 30 items, 2653 participants (2644 with 31 items and 9 patients with 30 items) were included for calculation of FI. Each participant received a score for each nonmissing item so that the FI score was calculated by summing the scores divided by their total number of nonmissing items. This approach minimises bias by ensuring that only participants with sufficient data were included and that missing data did not affect the FI calculation. Participants were divided into three groups: nonfrail (FI ≤0.210), moderately frail (FI 0.211–0.310) and severely frail (FI ≥0.311), based on thresholds used in previous studies [22, 29, 30].

### 2.3 Outcomes

The primary outcome defined in SAVE, and used in these analyses, was a composite of cardiovascular events that included death from any cardiovascular cause, myocardial infarction (including silent myocardial infarction), stroke, hospitalisation for heart failure, hospitalisation for an acute coronary syndrome (including unstable angina) and hospitalisation for a transient ischemic attack (TIA). Secondary outcomes were stroke, myocardial infarction, hospitalisation for unstable angina, all‐cause mortality, CVD death, composite of cerebral events, composite of cardiac events and revascularisation procedures [24, 26]. The primary and secondary cardiovascular outcome measures were prespecified in the SAVE trial protocol. All major cardiovascular outcomes were adjudicated by an independent clinical events committee whose members were blinded to treatment allocation [24, 26]. Poor adherence to CPAP was defined as an average use <4 h per night duration of the trial postrandomisation in the CPAP group [24].

### 2.4 Statistical Analysis

Baseline characteristics across FI groups (nonfrail, moderately frail and severely frail) were summarised as mean and standard deviation (SD) or median and interquartile interval (IQI), as appropriate, or frequency (%) and compared using ANOVA, Wilcoxon rank‐sum, or chi‐squared tests, as appropriate. Cumulative rates of the study outcomes across the three FI groups were illustrated using Kaplan–Meier curves and compared using log‐rank tests. Associations between FI and primary and secondary outcomes were assessed in Cox proportional hazards models that included adjustments for age, sex, region (non‐Asian vs. Asian), waist–hip ratio, ODI and randomised CPAP allocation. Results from Cox models are reported as hazard ratios (HRs) and 95% confidence intervals (CIs) for the three categories of FI (nonfrail as the reference) and per SD increment of FI. In addition, restricted cubic splines (with three knots at the 10th, 50th and 90th percentiles) were used to assess the shape of associations between the FI and the outcomes [31].

Tests of the homogeneity of the treatment effects of the CPAP randomised intervention on the outcomes according to the three FI groups were conducted by adding interaction terms in the multivariable‐adjusted Cox regression models. To assess the robustness of associations between the FI and the primary outcome, subgroup analyses were conducted in the multivariable‐adjusted models stratified by age (≥61 vs. <61 years), sex, region (Asian vs. non‐Asian), history of coronary artery disease, history of cerebrovascular disease, apnoea–hypopnea index (AHI; ≥30 vs. <30 events/h), ODI (≥24 vs. <24 events/h) and ESS score (0–9 vs. 10–15). Additionally, we conducted a sensitivity analysis using a 27‐item FI, excluding the history of the cardiovascular events at baseline (myocardial infarction, angina, heart failure, stroke, or TIA). All analyses were performed with Stata 17.0 (Stata Corp, College Station, TX, USA).

## 3 Results

Of the 2687 SAVE participants, a total of 2653 (mean age 61.3 [SD 7.8] years, 507 [19.1%] female and mean AHI 29.3 [SD 16.2]) with available data for calculating an FI were included in this study. The distribution of FI is shown in Figure S1. The mean and median FI were 0.290 (SD 0.125, range from 0.032 to 0.742) and 0.274 (IQI, 0.194–0.371), respectively. There were 864 (32.6%) nonfrail (FI ≤ 0.210), 783 (29.5%) moderately frail (FI 0.211–0.310) and 1006 (37.9%) severely frail (FI ≥ 0.311) participants. Table 1 shows the baseline characteristics of participants according to FI categories. In comparison to those with a lower FI, those with a higher FI tended to be female, non‐Asian and to have more CVD and other comorbidities. However, there were no significant differences in age and AHI across FI levels. In addition, there was no significant difference in CPAP intervention allocation across the three frailty groups. The distribution of FI in those with coronary artery disease and cerebrovascular disease is shown in Figure S2; the FI in patients with coronary artery disease (mean 0.313 [SD 0.126]) was higher than those with cerebrovascular disease (mean 0.269 [SD 0.120]).

**TABLE 1:** Baseline characteristics of participants by FI in SAVE.

Characteristics	Total	FI ≤ 0.210 (not frail)	FI 0.211–0.310 (moderately frail)	FI ≥ 0.311 (severely frail)	*p*
No. of patients	2653	864	783	1006	
Age, y	61.3 (7.8)	60.9 (8.0)	61.5 (7.9)	61.3 (7.6)	0.28
Gender					0.049
Female	507 (19.1)	166 (19.2)	129 (16.5)	212 (21.1)	
Male	2146 (80.9)	698 (80.8)	654 (83.5)	794 (78.9)	
Region					<0.001
Residence in Asia	1675 (63.2)	675 (78.1)	516 (65.9)	484 (48.2)	
Residence outside of Asia	978 (36.8)	189 (21.9)	267 (34.1)	522 (51.8)	
Medical and lifestyle history					
Hypertension	2080 (78.4)	526 (60.9)	656 (83.8)	898 (89.3)	<0.001
Diabetes	787 (29.7)	115 (13.3)	225 (28.7)	447 (44.4)	<0.001
Coronary artery disease	1453 (54.8)	375 (43.4)	437 (55.8)	641 (63.7)	<0.001
Cerebrovascular disease	1308 (49.3)	504 (58.3)	376 (48.0)	428 (42.5)	<0.001
Any stroke	1170 (44.1)	417 (48.3)	348 (44.4)	405 (40.3)	0.002
Any TIA	261 (9.8)	121 (14.0)	72 (9.2)	68 (6.8)	<0.001
Angina	980 (36.9)	212 (24.5)	293 (37.4)	475 (47.2)	<0.001
Myocardial infarction	890 (33.5)	200 (23.1)	276 (35.2)	414 (41.2)	<0.001
PCI or CABG	1146 (43.2)	260 (30.1)	355 (45.3)	531 (52.8)	<0.001
Valvular heart disease	47 (1.8)	7 (0.8)	12 (1.5)	28 (2.8)	0.005
Other CVD	115 (4.3)	17 (2.0)	35 (4.5)	63 (6.3)	<0.001
CAS, CEA or intracerebral stent	27 (1.0)	7 (0.8)	3 (0.4)	17 (1.7)	0.018
Heart failure	51 (1.9)	3 (0.3)	7 (0.9)	41 (4.1)	<0.001
Ever smoker	1480 (55.8)	370 (42.8)	463 (59.1)	647 (64.3)	<0.001
Alcohol consumption	679 (25.6)	210 (24.3)	217 (27.7)	252 (25.0)	0.25
Sedentary lifestyle^a^	422 (15.9)	44 (5.1)	101 (12.9)	277 (27.5)	<0.001
Body mass index, kg/m^2^	28.7 (4.5)	26.8 (3.2)	28.3 (3.8)	30.5 (5.2)	<0.001
Waist measurement (cm)	101.0 (12.2)	96.2 (10.9)	100.4 (10.7)	105.6 (12.5)	<0.001
Waist–hip ratio (cm/cm)	1.0 (0.1)	0.9 (0.1)	1.0 (0.1)	1.0 (0.1)	<0.001
Neck circumference	40.7 (4.1)	39.7 (3.9)	40.7 (3.9)	41.6 (4.2)	<0.001
Cardiovascular factors					
Systolic BP, mmHg	131.2 (16.1)	125.9 (11.9)	132.2 (15.5)	135.1 (18.2)	<0.001
Diastolic BP, mmHg	79.6 (10.7)	77.6 (9.3)	80.1 (10.6)	81.0 (11.7)	<0.001
Pulse pressure, mmHg	51.6 (12.8)	48.2 (10.1)	52.1 (13.2)	54.1 (14.0)	<0.001
Heart rate, bpm	71.3 (11.2)	69.8 (9.8)	71.2 (11.2)	72.6 (12.2)	<0.001
OSA characteristics					
ODI	28.2 (14.3)	26.3 (13.3)	28.2 (13.8)	29.9 (15.2)	<0.001
AHI	29.3 (16.2)	28.2 (15.5)	29.7 (16.1)	29.8 (16.8)	0.073
ESS score	7.4 (3.6)	6.2 (3.2)	7.3 (3.5)	8.5 (3.7)	<0.001
Randomised treatment					0.380
CPAP treatment	1327 (50.0)	430 (49.8)	407 (52.0)	490 (48.7)	
Usual care	1326 (50.0)	434 (50.2)	376 (48.0)	516 (51.3)	
Prior use of medications					
Polypharmacy (use ≥5 drugs)	228 (8.6)	15 (1.7)	50 (6.4)	163 (16.2)	<0.001
Antihypertensive agent	2074 (78.2)	550 (63.7)	654 (83.5)	870 (86.5)	<0.001
Statin or other lipid‐lowering agent	1552 (58.5)	401 (46.4)	456 (58.2)	695 (69.1)	<0.001
Antidiabetic oral medication	577 (21.7)	79 (9.1)	155 (19.8)	343 (34.1)	<0.001
Any insulin	163 (6.1)	10 (1.2)	35 (4.5)	118 (11.7)	<0.001
Aspirin or other antithrombotic agent	2003 (75.5)	551 (63.8)	617 (78.8)	835 (83.0)	<0.001
Depression/Anxiety					
HADS Depression	5.2 (3.9)	3.4 (3.0)	4.4 (3.2)	7.4 (4.0)	<0.001
HADS Anxiety	4.6 (3.6)	2.8 (2.3)	3.9 (2.9)	6.7 (4.0)	<0.001
SF‐36 8 domains					
SF‐36 Physical functioning	75.4 (20.7)	87.2 (10.6)	79.7 (16.3)	62.0 (22.6)	<0.001
SF‐36 Role limitations due to physical health	75.5 (26.3)	91.0 (14.1)	81.7 (20.4)	57.4 (27.7)	<0.001
SF‐36 Role limitations due to emotional problems	81.7 (24.3)	94.2 (11.3)	88.3 (16.8)	65.8 (28.5)	<0.001
SF‐36 Vitality	67.8 (20.2)	80.3 (12.5)	72.7 (14.9)	53.2 (20.0)	<0.001
SF‐36 Mental health	78.2 (17.4)	87.4 (9.7)	82.9 (12.4)	66.6 (19.3)	<0.001
SF‐36 Social functioning	80.0 (22.6)	91.4 (13.2)	86.0 (16.4)	65.7 (25.4)	<0.001
SF‐36 Body pain	78.0 (23.0)	90.2 (12.9)	82.8 (18.3)	63.8 (25.5)	<0.001
SF‐36 General health	52.0 (20.1)	62.1 (17.7)	53.9 (18.3)	41.9 (18.5)	<0.001

*Note:* Values are mean (SD) for continuous variables and number (%) for categorical variables.

Abbreviations: BP, blood pressure; CAS, carotid artery stenting; CABG, coronary artery bypass grafting; CEA, carotid endarterectomy; CPAP, continuous positive airway pressure; HADS, Hospital Anxiety and Depression Scale; PCI, percutaneous coronary intervention; SF‐36, 36‐Item Short Form Health Survey.

^a^ Sedentary is defined as engaging in less than 15 min per week of low, moderate and vigorous exercise.

Over a mean follow‐up of 3.7 years, 430 (16.2%) participants experienced a primary composite cardiovascular event. This included 132 (5.0%) strokes, 81 (3.1%) myocardial infarctions, 187 (7.1%) hospitalisations for unstable angina, 81 (3.1%) all‐cause deaths and 43 (1.6%) CVD deaths. Participants with severe frailty had a significantly higher cumulative rate of the composite cardiovascular endpoint (log‐rank *p* < 0.0001), strokes (log‐rank *p* = 0.0206), myocardial infarctions (log‐rank *p* = 0.0159), hospitalisations for unstable angina (log‐rank *p* < 0.0001), all‐cause mortality (log‐rank *p* = 0.0277) and CVD death (log‐rank *p* = 0.0278; Figure S3). Table 2 shows that, compared to the no frailty group, those with severe frailty had much higher rates of major cardiovascular events: composite cardiovascular events (HR, 2.41; 95% CI, 1.88–3.11; *P* trend < 0.001), stroke (HR, 2.40; 95% CI, 1.54–3.74; *P* trend < 0.001), hospitalisation for unstable angina (HR, 2.94; 95% CI, 1.98–4.35; *P* trend < 0.001), all‐cause mortality (HR, 1.77; 95% CI, 1.01–3.11; *P* trend = 0.04) and CVD death (HR, 2.51; 95% CI, 1.13–5.60; *P* trend = 0.02). Additionally, increases in FI (per SD) were significantly associated with a higher risk of composite cardiovascular events (HR, 1.50; 95% CI, 1.36–1.66), stroke (HR, 1.55; 95% CI, 1.30–1.86), hospitalisation for unstable angina (HR, 1.57; 95% CI, 1.36–1.82), all‐cause mortality (HR, 1.28; 95% CI, 1.03–1.61), CVD death (HR, 1.56; 95% CI, 1.15–2.12) as well as myocardial infarction (HR, 1.28; 95% CI, 1.03–1.59; Table 2). There were also significant associations between frailty and risks of composite cerebral events, composite cardiac events and revascularisation procedures (Table S2). In the sensitivity analysis using the 27‐item FI, which excluded baseline CVD history items, we found that a higher FI remained significantly associated with an increased risk of both composite and individual cardiovascular outcomes (Table S3).

**TABLE 2:** HRs (95% CIs) for the composite cardiovascular endpoint and for stroke, myocardial infarction, hospitalisation for unstable angina, all‐cause mortality and CVD death, according to the FI in SAVE.

	FI				
	≤0.210	0.211–0.310	≥0.311	*P* trend	Each 1‐SD increase in FI
Composite cardiovascular events					
Events, *n* (%)	96 (11.1)	126 (16.1)	208 (20.7)		
Unadjusted	1.00	1.63 (1.25–2.12)	2.32 (1.82–2.96)	<0.001	1.45 (1.32–1.58)
Adjusted	1.00	1.64 (1.25–2.15)	2.41 (1.88–3.11)	<0.001	1.50 (1.36–1.66)
Stroke					
Events, *n* (%)	32 (3.7)	41 (5.2)	59 (5.9)		
Unadjusted	1.00	1.54 (0.97–2.44)	1.83 (1.19–2.81)	0.01	1.30 (1.11–1.53)
Adjusted	1.00	1.71 (1.07–2.73)	2.40 (1.54–3.74)	<0.001	1.55 (1.30–1.86)
Myocardial infarction					
Events, *n* (%)	18 (2.1)	23 (2.9)	40 (4.0)		
Unadjusted	1.00	1.52 (0.82–2.81)	2.20 (1.26–3.84)	0.01	1.49 (1.21–1.83)
Adjusted	1.00	1.25 (0.67–2.33)	1.53 (0.85–2.73)	0.15	1.28 (1.03–1.59)
Hospitalisation for unstable angina					
Events, *n* (%)	37 (4.3)	55 (7.0)	95 (9.4)		
Unadjusted	1.00	1.82 (1.20–2.76)	2.68 (1.83–3.93)	<0.001	1.48 (1.29–1.69)
Adjusted	1.00	1.89 (1.24–2.87)	2.94 (1.98–4.35)	<0.001	1.57 (1.36–1.82)
All‐cause mortality					
Events, *n* (%)	20 (2.3)	22 (2.8)	39 (3.9)		
Unadjusted	1.00	1.33 (0.73–2.44)	1.99 (1.16–3.42)	0.01	1.37 (1.11–1.69)
Adjusted	1.00	1.27 (0.69–2.33)	1.77 (1.01–3.11)	0.04	1.28 (1.03–1.61)
CVD death					
Events, *n* (%)	9 (1.0)	11 (1.4)	23 (2.3)		
Unadjusted	1.00	1.48 (0.61–3.56)	2.59 (1.20–5.61)	0.01	1.59 (1.21–2.10)
Adjusted	1.00	1.43 (0.59–3.46)	2.51 (1.13–5.60)	0.02	1.56 (1.15–2.12)

*Note:* Adjusted for age, sex, region (non‐Asian vs. Asian), oxygen desaturation index, waist–hip ratio and CPAP allocation.

Abbreviations: CPAP, continuous positive airway pressure; CVD, cardiovascular disease; SD, standard deviation.

**FIGURE 1:**
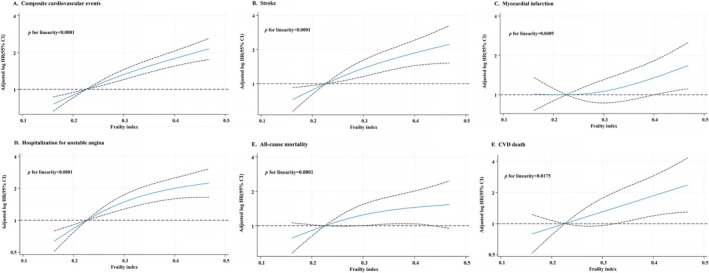
Relationship between FI and study outcomes in SAVE. (A) Composite cardiovascular events; (B) stroke; (C) myocardial infarction; (D) hospitalisation for unstable angina; (E) all‐cause mortality; (F) CVD death. HRs were estimated using Cox proportional hazards models with restricted cubic splines (knots at the 10th, 50th and 90th percentiles) to assess the shape of association between FI and cardiovascular outcomes. Abbreviations: CVD, cardiovascular disease; FI, frailty index; SAVE, Sleep Apnea Cardiovascular Endpoints.

Multivariable‐adjusted spline regression models showed a linear association between FI levels and composite cardiovascular events (*p* for linearity < 0.0001), stroke (*p* for linearity < 0.0001), hospitalisation for unstable angina (*p* for linearity < 0.0001), CVD death (*p* for linearity = 0.0175), myocardial infarctions (*p* for linearity = 0.0409) but not all‐cause mortality (*p* for linearity = 0.0801; Figure 1).

There was consistency in the associations between higher FI and increased risk of composite cardiovascular events across subgroups by age, region, sex, history of coronary artery disease, history of cerebrovascular disease and baseline AHI, ODI and ESS score (Table 3). There was evidence that the magnitude of the association between level of FI and higher risk of composite cardiovascular events was greater in those with a history of cerebrovascular disease (*p* = 0.045), but there was no significant interaction between FI and any other subgroup variable (Table 3).

**TABLE 3:** HRs (and 95% CIs) for the composite cardiovascular endpoint by FI in SAVE in subgroup analyses.

	FI				
	≤0.210	0.211–0.310	≥0.311	*P* trend	*P* for interaction
Region					0.709
Asian	1.00	1.94 (1.43–2.63)	2.59 (1.93–3.48)	<0.001	
non‐Asian	1.00	0.89 (0.51–1.58)	1.67 (1.02–2.73)	0.01	
Sex					0.091
Male	1.00	1.44 (1.08–1.91)	2.18 (1.66–2.85)	<0.001	
Female	1.00	3.60 (1.63–7.95)	4.26 (1.97–9.22)	<0.001	
Age, years					0.741
<61	1.00	1.63 (1.09–2.46)	2.73 (1.87–3.98)	<0.001	
≥61	1.00	1.66 (1.16–2.37)	2.23 (1.59–3.13)	<0.001	
History of coronary artery disease					0.581
No	1.00	1.82 (1.21–2.73)	2.56 (1.73–3.78)	<0.001	
Yes	1.00	1.40 (0.98–1.99)	2.11 (1.51–2.94)	<0.001	
History of cerebrovascular disease					0.045
No	1.00	1.33 (0.91–1.94)	1.79 (1.25–2.57)	0.001	
Yes	1.00	1.92 (1.31–2.80)	3.13 (2.20–4.45)	<0.001	
AHI, events/h					0.478
<30	1.00	1.60 (1.15–2.22)	2.26 (1.65–3.09)	<0.001	
≥30	1.00	1.80 (1.13–2.88)	2.75 (1.78–4.25)	<0.001	
Oxygen desaturation index (4% events/h)					0.107
<24	1.00	1.54 (1.09–2.19)	2.00 (1.43–2.80)	<0.001	
≥24	1.00	1.86 (1.22–2.83)	2.98 (2.01–4.41)	<0.001	
ESS score					0.185
0–9	1.00	1.61 (1.20–2.16)	2.36 (1.78–3.13)	<0.001	
10–15	1.00	2.51 (1.14–5.51)	4.10 (1.96–8.56)	<0.001	

*Note:* All models were adjusted for the same covariates as in the fully adjusted model presented in Table 2, except for the stratification variable itself.

Abbreviations: CPAP, continuous positive airway pressure.

**FIGURE 2:**
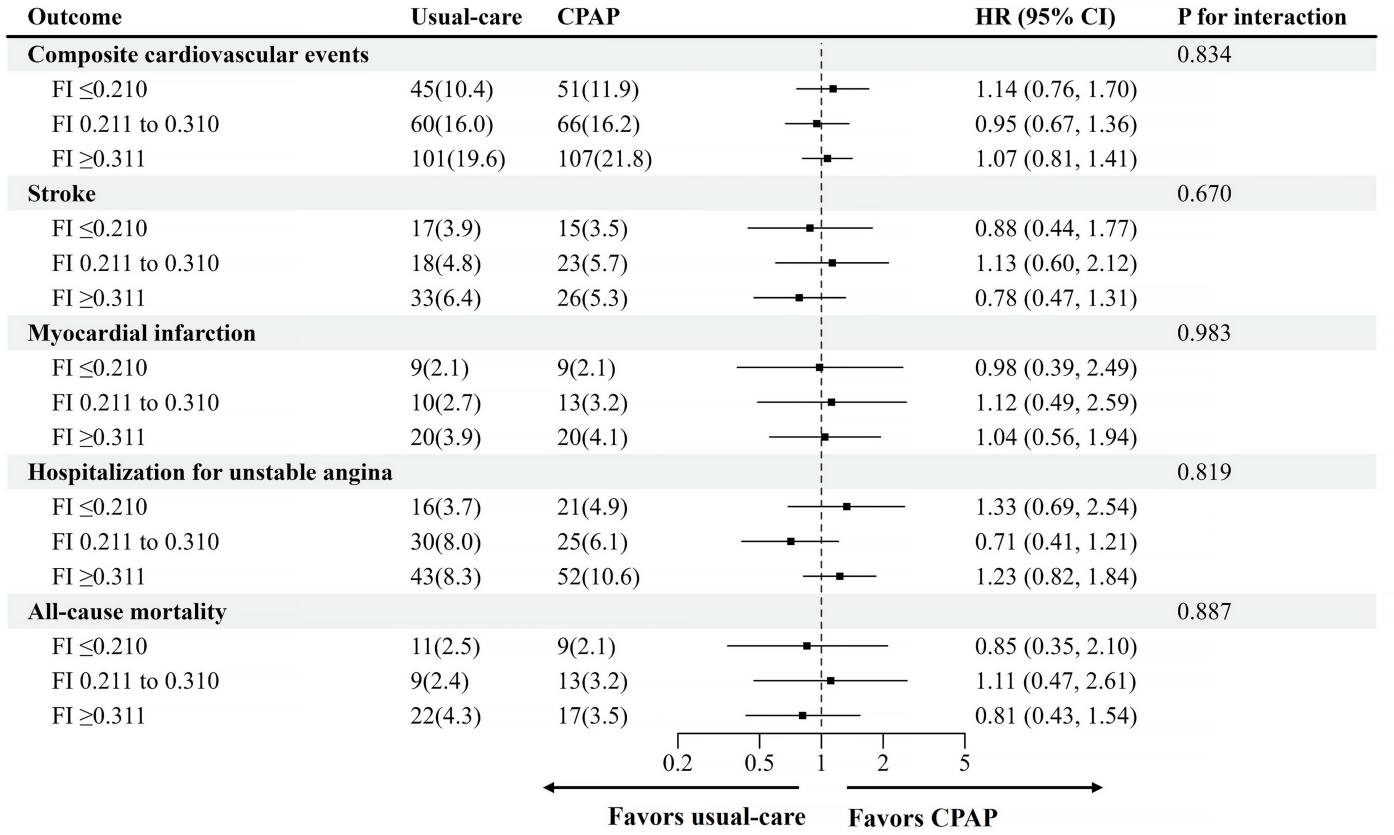
HRs (95% CIs) of the as‐randomised CPAP treatment effect according to FI. Adjusted for age, sex, region (non‐Asian vs. Asian), oxygen desaturation index and waist–hip ratio. Abbreviations: CPAP, continuous positive airway pressure; FI, frailty index.

Outcomes of composite cardiovascular events, stroke, hospitalisation for unstable angina and all‐cause mortality were not significantly different between the CPAP plus usual care group and the usual care group, according to the subgroups by baseline FI (Figure 2). In addition, there was no evidence of heterogeneity in the effect of CPAP treatment by FI on composite cardiovascular events (*p* = 0.834 for interaction), stroke (*p* = 0.670 for interaction), myocardial infarction (*p* = 0.983 for interaction), hospitalisation for unstable angina (*p* = 0.819 for interaction) and all‐cause mortality (*p* = 0.887 for interaction; Figure 2).

We found no significant difference in CPAP adherence in the nonfrail, moderately frail and severely frail participants (*p* = 0.488). Poor adherence was recorded in 268 (62.5%), 236 (59.2%) and 284 (58.9%) participants, respectively (Figure S4).

## 4 Discussion

This post hoc analysis of the SAVE study revealed that frailty (FI >0.21) affected approximately two‐thirds of participants who had moderate‐to‐severe OSA and established coronary artery disease and/or cerebrovascular disease. Participants with greater frailty were at higher risk of composite and individual cardiovascular events including stroke, hospitalisation for unstable angina, all‐cause mortality and CVD death. The associations between greater frailty and increased risk of composite cardiovascular events were consistent across subgroups defined by age, region, sex, history of coronary artery disease or cerebrovascular disease and baseline OSA severity. There was no heterogeneity in the effect of CPAP treatment on CVD outcomes by baseline frailty status, and frailty did not influence CPAP adherence.

The frequency of frailty varies across studies according to definition and population setting [21]. Data from the China Kadoorie Biobank study found that 3.1% of 512 723 community‐dwelling adults were frail (FI ≥0.25) [14], whereas a trial in heart failure patients found that 59% of participants were frail (FI >0.21) [23]. Among those with stroke, three international population surveys found that 54.6% of patients had moderate or severe frailty [16] and an Australian myocardial infarction registry study reported frailty in 27.7% of participants (FI >0.25) [17]. In our study of patients with OSA and established CVD, participants had higher FI compared to previous studies [14, 15, 16, 17, 21, 23]. The mean FI was 0.290 (ranging from 0.032 to 0.742), with 67.4% of patients experiencing frailty—29.5% classified as moderately frail (FI: 0.211–0.310) and 37.9% as severely frail (FI ≥0.311). The high rate of frailty in our study is not surprising, given that the SAVE participants have coexisting moderate‐to‐severe OSA and established CVD. Recent studies indicate that people with OSA are more likely to have frailty [15, 32]. Moreover, the OSA population generally have more cardiovascular risk factors and comorbidities compared to those without OSA [6, 25, 33].

Previous studies indicate that higher FI increases the risks of cardiovascular events and all‐cause mortality in the community and in patients with hypertension or diabetes mellitus [14, 18, 19, 34]. Studies in stroke and myocardial infarction have also found that an increased FI is associated with higher risks of all‐cause mortality, incident hospitalisation and disability on follow‐up [16, 17, 35, 36]. However, data on FI and recurrent cardiovascular events are limited. In our current analyses, we found that higher FI was significantly associated with an increased risk of composite and individual cardiovascular events and all‐cause mortality in OSA patients with established CVD. Our findings therefore add to the literature by showing a clear dose‐dependent relationship between FI and recurrent cardiovascular events. Current literature suggests that pathological mechanisms underlying the relationship between frailty and cardiovascular risk may involve systemic inflammation, endothelial dysfunction and autonomic dysregulation [3, 5].

In the subgroup analysis, the association between the frailty status and the risk of composite cardiovascular events was stronger in individuals with a history of cerebrovascular disease than in those without baseline cerebrovascular disease (those patients with previous coronary artery disease). Although this could simply be a chance finding, a possible explanation is that in the SAVE dataset, the FI was higher in participants with coronary artery disease (mean 0.313) than in those with cerebrovascular disease (mean 0.269). Because most patients with coronary artery disease were already highly frail, the impact of FI on the risk of composite cardiovascular events may thus have been less pronounced in this group. We found that higher FI scores and an increased risk of composite cardiovascular events were consistent across age groups (<61 vs. ≥61 years). Recent studies have also shown that frailty significantly increases the risk of mortality in younger and middle‐aged populations, as well as in patients with myocardial infarction [36, 37]. Together with previous evidence, our findings indicate that frailty should be assessed in young and middle‐aged adults.

Whether frailty influences adherence and outcome of CPAP treatment has been a controversial issue. Some studies indicate that adults with frailty‐associated comorbidity and polypharmacy have a reduced tolerance to treatments, poor adherence and are less likely to benefit from interventions [20, 21, 34, 38]. Conversely, studies in patients with heart failure and hypertension show that equal or even greater benefit of interventions in those with frailty [18, 22, 23, 29, 39, 40]. In the current analysis, we found frailty did not modify the effect of CPAP treatment: compared to usual care alone, CPAP in addition to usual care did not alter the risk of recurrent cardiovascular events across the levels of FI. CPAP adherence is a key factor influencing the secondary prevention effect of CPAP on cardiovascular events. An individual participant data meta‐analysis of three trials, including the SAVE study, indicated that good adherence to CPAP (≥4 h/per night) was significantly associated with a lower risk of recurrent major adverse cardiac and cerebrovascular events [41]. In the current analysis, we found baseline FI did not affect CPAP adherence. Our findings therefore indicate that CPAP treatment is equally suitable for frail adults with OSA because frailty did not modify the effect of CPAP treatment on cardiovascular events as well as CPAP adherence. Moreover, these findings suggest that frailty does not explain the neutral cardiovascular results of the SAVE trial.

### 4.1 Study Advantages and Limitations

Key strengths of our study include the use of the SAVE study dataset, the largest randomised clinical trial of CPAP in OSA participants with co‐occurring CVD who were well characterised, systematically assessed and followed up in various health care settings. To the best of our knowledge, this study is the first to construct an FI and establish the relationship of FI and recurrent cardiovascular events in patients with OSA and established CVD, and to show that FI did not modify the effect of CPAP.

There were some limitations to our study. First, as participants had moderate‐to‐severe OSA with existing CVD for inclusion in the SAVE study, the results may not be generalisable to adults with mild OSA or the broader OSA population without CVD. Second, we were unable to validate our findings using alternative frailty instruments such as the Fried physical frailty phenotype, which is based on measures including grip strength and gait speed as these data were unavailable in SAVE. Third, due to the observational and post hoc nature of our analyses using clinical trial data, causality cannot be inferred from these results. Moreover, the original trial design limited the availability and timing of certain variables and precluded real‐time modification of data collection. Fourth, we were unable to assess changes in frailty during the study due to the lack of some follow‐up data and a relatively short follow‐up period. In addition, frailty‐specific outcomes such as functional decline or disability were not systematically collected in SAVE, precluding formal validation of the FI against these endpoints. Finally, despite inclusion of important confounders in the multivariable‐adjusted models, the possibility of residual confounding cannot be fully eliminated in a post hoc study and direct evidence linking frailty to specific pathophysiological processes in our study is limited.

## 5 Conclusions

In summary, our analysis of the SAVE study indicates that frailty is common in adults with OSA and established CVD and is an independent predictor of death and recurrent cardiovascular events. However, frailty did not affect CPAP adherence or modify the effect of CPAP. Our findings re‐emphasise the importance of cardiovascular risk factor management in adults with OSA and established CVD, particularly those with features of frailty. Future studies to investigate strategies targeting frailty to improve outcomes are warranted.

## Author Contributions

Drs S.Y., D.Z. and C.A. contributed to the concept and rationale for the study. S.Y. conducted statistical analyses. S.Y. and D.Z. were responsible for the first draft; C.A., K.L., R.M., K.H., R.P., M.W., J.C. and Q.L. for major revisions. All authors participated in the review and approval of the final manuscript and take responsibility for its content and interpretation.

## Funding

The SAVE trial was supported by project grants (1006501 (2011–2015) and 1060078 (2014–2016)) from the National Health and Medical Research Council (NHMRC) of Australia and by Respironics Sleep and Respiratory Research Foundation and Philips Respironics. Supplementary trial funding was provided by Fisher & Paykel Healthcare, the Australasian Sleep Trials Network (enabling grant 343020 from the NHMRC), the Spanish Respiratory Society (grant 105‐2011) and Fondo de Investigaciones Sanitarias (grant 13/02053). In‐kind donations were provided by Respironics for CPAP equipment and by ResMed for sleep apnoea diagnostic devices.

## Consent

The authors have nothing to report.

## Conflicts of Interest

Dr. You holds the National Natural Science Foundation of China (82471226), Discipline Construction Program of the Second Affiliated Hospital of Soochow University (XKTJ‐RC202412), Jiangsu Provincial Medical Key Discipline (ZDXK202217) and the 6th Jiangsu Province 333 High Level Talents Training Project. Dr. Chalmers reports research grants from the NHMRC. Dr. Woodward also reports research support from the NHMRC, as well as consultancy work for Freeline in the recent past. Dr. Ferran Barbé is supported by the ICREA Academia program from Generalitat de Catalunya. Dr. Anderson holds a Senior Investigator Fellowship of the NHMRC and reports receipt of advisory board fees from AstraZeneca, Australia. Dr. Loffler reports grant funding from the Medical Research Future Fund, the American Academy of Sleep Medicine and Flinders Foundation, as well as contract research funding from Addend AI, unrelated to this project.

## Ethics Approval and Consent to Participate

Local institutional review boards or independent ethics committees at recruiting sites approved the protocol, and all participants provided written informed consent.

## Supporting information


**Table S1:** Items included in the FI.
**Table S2:** HRs (95% CIs) for a composite of cerebral events, a composite of cardiac events and revascularisation procedures according to the FI in SAVE.
**Table S3:** Sensitivity analysis: HRs (95% CIs) for composite and individual cardiovascular outcomes based on the 27‐item FI (excluding baseline CVD history items).
**Figure S1:** The distribution of the FI in SAVE.
**Figure S2:** The distribution of the FI in patients with baseline coronary artery disease and cerebrovascular disease.
**Figure S3:** Kaplan–Meier curves for cardiovascular events by FI in SAVE.
**Figure S4:** CPAP adherence duration over the trial period across baseline FI groups.

## Data Availability

Deidentified individual participant data used in these analyses may be shared by investigators upon request following approval of a protocol and signed data access agreement via the Research Office of The George Institute for Global Health, Australia.
