# Supplementary material for: Frailty and Recurrent Cardiovascular Events in Patients With Obstructive Sleep Apnoea: The SAVE Study
Source: J Cachexia Sarcopenia Muscle. 2026 Mar 19;17(2):e70252. doi: 10.1002/jcsm.70252 (PMC13140912; doi:10.1002/jcsm.70252)
Supplement: Supplementary file 1 — Table S1: Items included in the FI. Table S2: HRs (95% CIs) for a composite of cerebral events, a composite of cardiac events and revascularisation procedures according to the FI in SAVE. Table S3: Sensitivity analysis: HRs (95% CIs) for composite and individual cardiovascular outcomes based on the 27‐item FI (excluding baseline CVD history items). Figure S1: The distribution of the FI in SAVE. Figure S2: The distribution of the FI in patients with baseline coronary artery disease and cerebrovascular disease. Figure S3: Kaplan–Meier curves for cardiovascular events by FI in SAVE. Figure S4: CPAP adherence duration over the trial period across baseline FI groups. [file JCSM-17-e70252-s001.docx]

**ONLINE-ONLY SUPPLEMENT**

**Shoujiang You, et al. Frailty and recurrent cardiovascular events in patients with obstructive sleep apnea: the SAVE study**

**Contents**

Table S1. Items included in the frailty index.

Table S2. HRs (95% CIs) for a composite of cerebral events, a composite of cardiac events and revascularization procedures according to the Frailty Index in SAVE.

Table S3. Sensitivity Analysis: Hazard ratios (95% confidence intervals) for composite and individual cardiovascular outcomes based on the 27-item FI (excluding baseline CVD history items).

Figure S1. The distribution of the Frailty Index in SAVE.

Figure S2. The distribution of the Frailty Index in patients with baseline coronary artery disease and cerebrovascular disease.

Figure S3. Kaplan–Meier curves for cardiovascular events by frailty index in SAVE.

Figure S4. CPAP adherence duration over the trial period across baseline Frailty Index groups.

**Table S1. Items included in the frailty index.**

|  | **Items** | **Definition** | **Score** | **Numbers** | **Missing** |
| --- | --- | --- | --- | --- | --- |
|  | ***Comorbidities (*medical history*)*** | |  |  |  |
| **1** | Myocardial infarction | No | 0 | 1782 (66.5%) | 6(0.22%) |
|  |  | Yes | 1 | 899 (33.5%) |  |
| **2** | Hypertension | No | 0 | 578 (21.6%) | 6(0.22%) |
|  |  | Yes | 1 | 2103 (78.4%) |  |
| **3** | Diabetes | No | 0 | 1883 (70.2%) | 6(0.22%) |
|  |  | Yes | 1 | 798 (29.8%) |  |
| **4** | Angina | No | 0 | 1692 (63.1%) | 6(0.22%) |
|  |  | Yes | 1 | 989 (36.9%) |  |
| **5** | Heart failure | No | 0 | 2630 (98.1%) | 6(0.22%) |
|  |  | Yes | 1 | 51 (1.9%) |  |
| **6** | PCI or CABG | No | 0 | 1523 (56.8%) | 6(0.22%) |
|  |  | Yes | 1 | 1158 (43.2%) |  |
| **7** | Stroke or TIA | No | 0 | 1313 (49.0%) | 6(0.22%) |
|  |  | Yes | 1 | 1368 (51.0%) |  |
| **8** | Valvular heart diseases | No | 0 | 2633 (98.2%) | 6(0.22%) |
|  |  | Yes | 1 | 48 (1.8%) |  |
| **9** | CAS, CEA or Intracerebral stent | No | 0 | 2654 (99.0%) | 6(0.22%) |
|  |  | Yes | 1 | 27 (1.0%) |  |
| **10** | Other heart diseases | No | 0 | 2564 (95.6%) | 6(0.22%) |
|  |  | Yes | 1 | 117 (4.4%) |  |
| **11** | Polypharmacy (≥5 drugs at baseline) | No | 0 | 2457 (91.4%) | 0 |
|  |  | Yes | 1 | 230 (8.6%) |  |
|  | ***Anxiety or depression* (baseline measure of HADS)** | |  |  |  |
| **12** | Anxiety | HADS anxiety score ≥0 and ≤7 | 0 | 2150 (80.3%) | 10(0.37%) |
|  |  | HADS anxiety score ≥8 and ≤10 | 0.5 | 324 (12.1%) |  |
|  |  | HADS anxiety score ≥11 | 1 | 203 (7.6%) |  |
| **13** | Depression | HADS depression score ≥0 and ≤7 | 0 | 1968 (73.5%) | 10(0.37%) |
|  |  | HADS depression score ≥8 and ≤10 | 0.5 | 419 (15.7%) |  |
|  |  | HADS depression score ≥11 | 1 | 290 (10.8%) |  |
|  | ***Vital signs*** (cut-off point at 75th percentile) | |  |  |  |
| **14** | Systolic blood pressure (SBP) | < 140 mmHg | 0 | 1937 (72.4%) | 13(0.48%) |
|  |  | ≥ 140 mmHg | 1 | 737 (27.6%) |  |
| **15** | Diastolic blood pressure (DBP) | < 86 mmHg | 0 | 1974 (73.8%) | 13(0.48%) |
|  |  | ≥ 86 mmHg | 1 | 700 (26.2%) |  |
| **16** | Pulse pressure (SBP-DBP) | < 58.5 mmHg | 0 | 1987 (74.3%) | 13(0.48%) |
|  |  | ≥ 58.5 mmHg | 1 | 687 (25.7%) |  |
| **17** | Heart rate | < 78 bpm | 0 | 1925 (72.0%) | 13(0.48%) |
|  |  | ≥ 78 bpm | 1 | 749 (28.0%) |  |
|  | ***Lifestyle factors*** |  |  |  |  |
| **18** | Ever smoked | No | 0 | 1190 (44.4%) | 6(0.22%) |
|  |  | Yes | 1 | 1491 (55.6%) |  |
| **19** | Higher or lower BMI | BMI≥18.5 and <25 | 0 | 509 (19.0%) | 0 |
|  |  | BMI≥25 and <30 | 0.5 | 1314 (49.1%) |  |
|  |  | BMI≥30 or <18.5 | 1 | 855 (31.9%) |  |
| **20** | Sedentary (defined as low exercise >15 min per week=0 and moderate exercise >15 min per week=0 and vigorous exercise >15 min per week=0) | No | 0 | 2253 (84.1%) | 9(0.33%) |
|  |  | Yes | 1 | 425 (15.9%) |  |
| **21** | Higher neck circumference (use the cut-off point of 75th percentile) | < 43 cm | 0 | 1832 (68.7%) | 22(0.81%) |
|  |  | ≥ 43 cm | 1 | 833 (31.3%) |  |
|  | ***OSA severity measures*** | |  |  |  |
| **22** | Higher AHI | < 30 | 0 | 1588 (59.1%) | 0 |
|  |  | ≥ 30 | 1 | 1099 (40.9%) |  |
| **23** | Higher ESS | < 10 | 0 | 1894 (70.5%) | 0 |
|  |  | ≥ 10 | 1 | 793 (29.5%) |  |
|  | ***Eight domain Quality of life SF36*** (all use the cut-off point of 25th percentile) | | | | |
| **24** | Poor physical functioning | >65 | 0 | 1948 (73.0%) | 20(0.74%) |
|  |  | ≤65 | 1 | 719 (27.0%) |  |
| **25** | Role limitations due to physical health | >56.25 | 0 | 1959 (73.5%) | 20(0.74%) |
|  |  | ≤56.25 | 1 | 708 (26.5%) |  |
| **26** | Role limitations due to emotional problems | > 66.67 | 0 | 1990 (74.6%) | 20(0.74%) |
|  |  | ≤66.67 | 1 | 677 (25.4%) |  |
| **27** | Low vitality | >56.25 | 0 | 1867 (70.0%) | 20(0.74%) |
|  |  | ≤56.25 | 1 | 800 (30.0%) |  |
| **28** | Poor mental health | >70 | 0 | 1880 (70.5%) | 20(0.74%) |
|  |  | ≤70 | 1 | 787 (29.5%) |  |
| **29** | Poor social functioning | > 75 | 0 | 1493 (56.0%) | 20(0.74%) |
|  |  | ≤75 | 1 | 1174 (44.0%) |  |
| **30** | Have body pain | >67.5 | 0 | 1842 (69.1%) | 20(0.74%) |
|  |  | ≤67.5 | 1 | 825 (30.9%) |  |
| **31** | Poor general health | >35 | 0 | 1990 (74.6%) | 20(0.74%) |
|  |  | ≤35 | 1 | 677 (25.4%) |  |

Abbreviations: AHI, Apnea–hypopnea index; BMI, body mass index; CAS, carotid artery stenting; CABG, coronary artery bypass grafting; CEA, carotid endarterectomy; DBP, Diastolic blood pressure; ESS, Epworth Sleepiness Scale; HADS, Hospital Anxiety and Depression Scale; PCI, percutaneous coronary intervention; OSA, obstructive sleep apnea; SBP, Systolic blood pressure; SF36, 36-item Short Form Health Survey; TIA, transient ischemic attack.

**Table S2. HRs (95% CIs) of a composite of cerebral events, a composite of cardiac events and revascularization procedures according to the Frailty Index in SAVE.**

|  | **Frailty Index** | | | | |  | **Each 1-SD increase of**  **Frailty Index** |
| --- | --- | --- | --- | --- | --- | --- | --- |
|  | **≤0.210** | **0.211 to 0.310** | **≥0.311** | ***P* trend** |  | | |
| **Composite of cerebral events** | |  |  |  |  | |  |
| Events, n (%) | 39 (4.5) | 44 (5.6) | 67 (6.7) |  |  | |  |
| Unadjusted | 1.00 | 1.35 (0.88-2.08) | 1.71 (1.15-2.53) | 0.008 |  | | 1.28 (1.10-1.49) |
| Adjusted | 1.00 | 1.47 (0.95-2.28) | 2.14 (1.43-3.22) | <0.001 |  | | 1.48 (1.25-1.74) |
| **Composite of cardiac events** | |  |  |  |  | |  |
| Events, n (%) | 65 (7.5) | 97 (12.4) | 160 (15.9) |  |  | |  |
| Unadjusted | 1.00 | 1.84 (1.34-2.52) | 2.61 (1.95-3.48) | <0.001 |  | | 1.50 (1.35-1.66) |
| Adjusted | 1.00 | 1.76 (1.28-2.41) | 2.41 (1.78-3.25) | <0.001 |  | | 1.47 (1.32-1.64) |
| **Revascularization procedures** | |  |  |  |  | |  |
| Events, n (%) | 36 (4.2) | 55 (7.0) | 82 (8.2) |  |  | |  |
| Unadjusted | 1.00 | 1.85 (1.22-2.82) | 2.30 (1.55-3.41) | <0.001 |  | | 1.37 (1.19-1.57) |
| Adjusted | 1.00 | 1.72 (1.13-2.64) | 2.11 (1.40-3.17) | <0.001 |  | | 1.33 (1.14-1.54) |

Adjusted for age, sex, region (non-Asian vs. Asian), oxygen desaturation index, waist-hip ratio, and CPAP allocation.

Abbreviations: CPAP, continuous positive airway pressure; SD, standard deviation.

**Table S3. Sensitivity Analysis: Hazard ratios (95% confidence intervals) for composite and individual cardiovascular outcomes based on the 27-item FI (excluding baseline CVD history items).**

| **Outcomes** | **Unadjusted HR**  **(Each 1-SD increase in FI)** | **P value** | **Adjusted HR**  **(Each 1-SD increase in FI)** | **P value** |
| --- | --- | --- | --- | --- |
| Composite cardiovascular events | 1.40 (1.28-1.53) | <0.001 | 1.45 (1.31-1.59) | <0.001 |
| Stroke | 1.29 (1.10-1.52) | 0.002 | 1.53 (1.29-1.83) | <0.001 |
| Myocardial infarction | 1.44 (1.17-1.76) | <0.001 | 1.24 (1.00-1.54) | 0.054 |
| Hospitalization for unstable angina | 1.40 (1.23-1.60) | <0.001 | 1.48 (1.29-1.71) | <0.001 |
| All-cause mortality | 1.32 (1.07-1.63) | 0.009 | 1.24 (0.99-1.56) | 0.055 |
| CVD death | 1.52 (1.15-2.01) | 0.003 | 1.49 (1.10-2.02) | 0.010 |

Adjusted for age, sex, region (non-Asian vs. Asian), oxygen desaturation index, waist-hip ratio, and CPAP allocation.

Abbreviations: CPAP, continuous positive airway pressure; SD, standard deviation.

**Figure S1. The distribution of the Frailty Index in SAVE.**


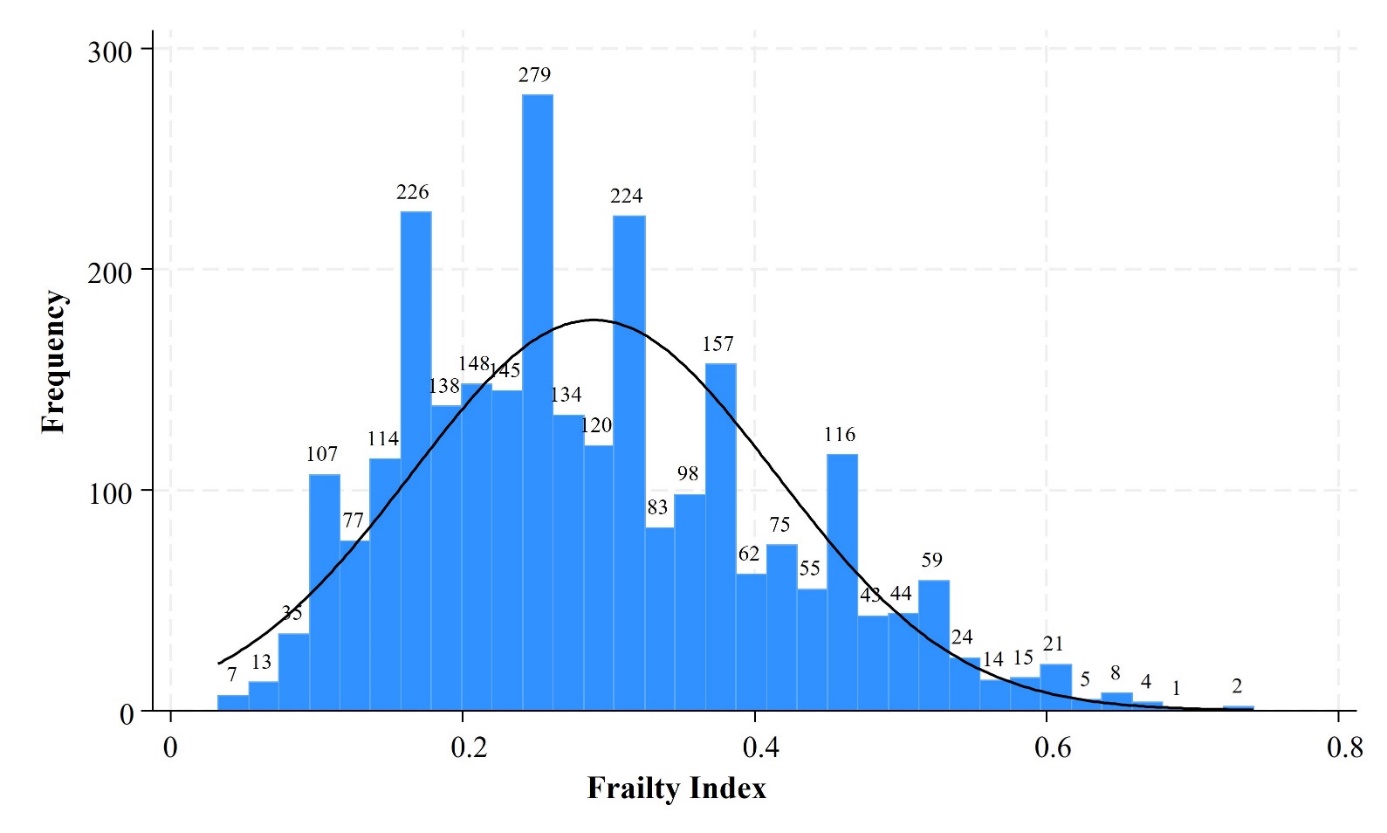


Abbreviations: OSA, obstructive sleep apnea; SAVE, Sleep Apnea Cardiovascular Endpoints.

**Figure S2. The distribution of the Frailty Index in patients with baseline coronary artery disease and cerebrovascular disease.**


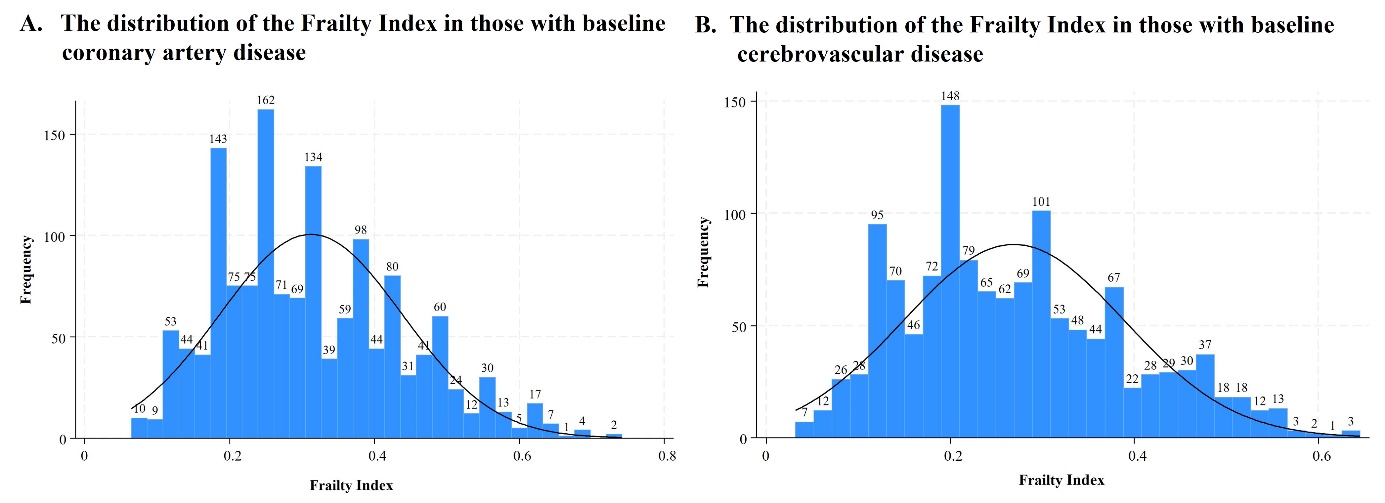


**Figure S3. Kaplan–Meier curves for cardiovascular events by frailty index in SAVE.**


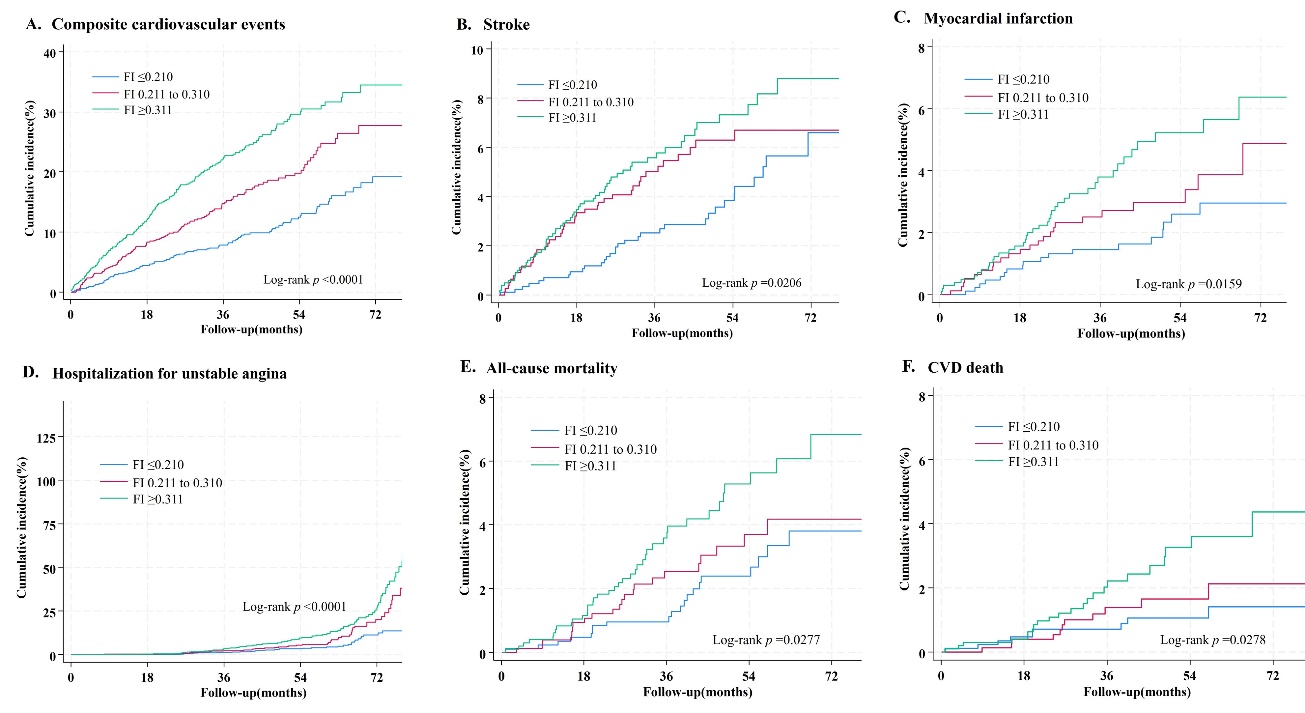


1. Composite cardiovascular events; (B) Stroke; (C) Myocardial infarction;

(D) Hospitalization for unstable angina; (E) All-cause mortality; (F) CVD death.

**Figure S4. The CPAP adherence duration of the trial among baseline Frailty Index.**


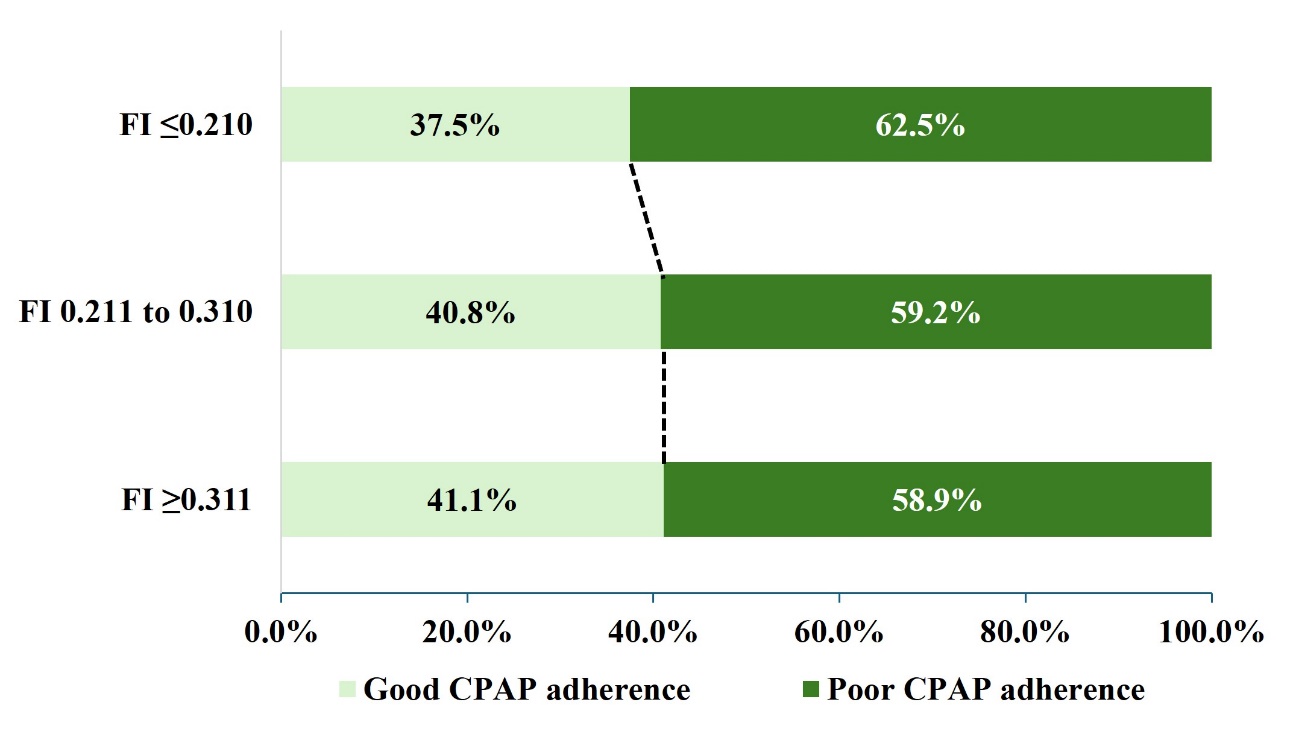


Good and poor CPAP adherence defined as the average use of CPAP≥4 hours, <4 hours

per night duration of the trial among CPAP group, respectively.
